# Affect and the Brain's Functional Organization: A Resting-State Connectivity Approach

**DOI:** 10.1371/journal.pone.0068015

**Published:** 2013-07-23

**Authors:** Christiane S. Rohr, Hadas Okon-Singer, R. Cameron Craddock, Arno Villringer, Daniel S. Margulies

**Affiliations:** 1 Department of Neurology, Max Planck Institute for Human Cognitive and Brain Sciences, Leipzig, Germany; 2 Mind-Brain Institute, Berlin School of Mind and Brain, Humboldt University, Berlin, Germany; 3 Child Mind Institute, New York, New York, United States of America; 4 Nathan S. Kline Institute for Psychiatric Research, Orangeburg, New York, United States of America; Institute of Psychology, Chinese Academy of Sciences, China

## Abstract

The question of how affective processing is organized in the brain is still a matter of controversial discussions. Based on previous initial evidence, several suggestions have been put forward regarding the involved brain areas: (a) right-lateralized dominance in emotional processing, (b) hemispheric dominance according to positive or negative valence, (c) one network for all emotional processing and (d) region-specific discrete emotion matching. We examined these hypotheses by investigating intrinsic functional connectivity patterns that covary with results of the Positive and Negative Affective Schedule (PANAS) from 65 participants. This approach has the advantage of being able to test connectivity rather than activation, and not requiring a potentially confounding task. Voxelwise functional connectivity from 200 regions-of-interest covering the whole brain was assessed. Positive and negative affect covaried with functional connectivity involving a shared set of regions, including the medial prefrontal cortex, the anterior cingulate, the visual cortex and the cerebellum. In addition, each affective domain had unique connectivity patterns, and the lateralization index showed a right hemispheric dominance for negative affect. Therefore, our results suggest a predominantly right-hemispheric network with affect-specific elements as the underlying organization of emotional processes.

## Introduction

The breadth of research on how the brain processes emotions has led to several prominent theories of the underlying large-scale functional systems.

**Figure 1:**
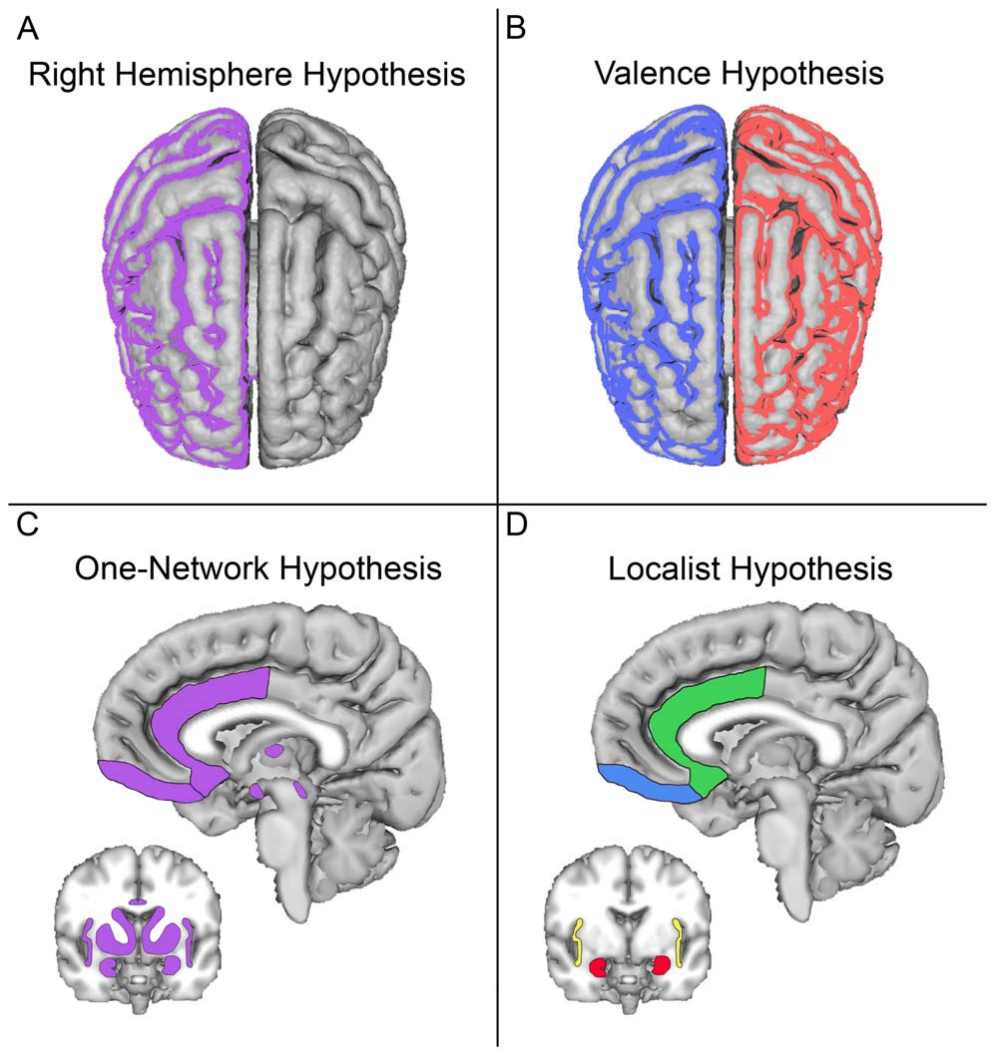
Four hypotheses for emotion processing in the brain have been put forward. (Figure based on [15]. The right hemisphere hypothesis (a) assumes that emotion is processed predominantly in the right hemisphere. The valence hypothesis (b) suggests the right hemisphere to be dominant in processing negative emotions and the left hemisphere to be dominant in processing positive emotions. The one-network hypothesis (c) posits that all emotions may be processed by a set of brain regions not specific to a respective emotion category, while the localist hypothesis (d) is that processing of different emotions specifically corresponds to activation in distinct brain regions.

One prominent theory asserts that emotion is processed predominantly in the right hemisphere (Figure 1A), while the left hemisphere is primarily involved in cognitive processes. This hypothesis is supported by behavioral studies showing that visual stimuli processed in the right hemisphere (presented to the left visual field) are judged as more emotional [1] and emotional intonation is more easily recognized when presented to the left ear [2]. Patients with lesions to the right hemisphere have also been found to have greater impairment in the perception of emotionally expressive faces as compared to patients with comparable lesions to the left hemisphere [3].

A second theory regarding locations of affective processing suggests that both hemispheres are involved, but each is primarily concerned with different types of emotion: the right hemisphere is suggested to be dominant in processing negative emotions, whereas the left hemisphere is suggested to be dominant in processing positive emotions (Figure 1B). This hypothesis is supported by behavioral studies showing that positive emotions are potentiated when stimuli are presented to the left hemisphere, and negative emotions are potentiated when presented to the right hemisphere [4], [5]. Here again, lesion studies have found that damage to the left hemisphere impaired the perception of positive emotions, while comparable lesions to the right hemisphere impaired the perception of negative emotions [6]. Moreover, a disproportionate number of patients who have suffered trauma to the left frontal lobe, especially the lateral prefrontal cortex or basal ganglia, have become depressed [7], [8]; in contrast, patients with right frontal damage were more likely to show signs of inappropriate cheerfulness and mania [9]. Further support for this hypothesis also comes from a set of EEG experiments conducted by Davidson and colleagues [10]–[13] who proposed that this lateralization might be particularly dominant in the frontal lobe.

A third theory moves beyond just lateralization, suggesting that all emotions are processed by a specific set of brain regions across all categories of emotion (Figure 1C). Although these differ with regard to details, they all share the belief that affective processing relies on cognitive systems serving a variety of functions. Examples would be the “salience network” approach, where the salience network interlinks with an executive control network [14] or the “constructionist” hypothesis [15], which suggests a network of regions concerned with four operations: conceptualization, executive attention, language and core affect.

Yet a fourth theory posits that the processing of different emotions corresponds to activation in distinct sets of brain regions (Figure 1D). Models falling within this “localist” theory typically assume that discrete emotions form the smallest psychological entities at play, which cannot be broken up any further. Studies have found single brain regions to play crucial roles in either positive or negative distinct emotions: the amygdala activated in response to fear [16]–[18], the insula to disgust [19], the subgenual anterior cingulate cortex to sadness [20], [21], and the basal ganglia to happiness [17], [18].

One novel way to explore these four views is through investigating how affective processing relates to network organization. Intrinsic functional connectivity provides a means to investigate brain networks, and has yielded reliable results in correlating self-report measures of personality traits to functional patterns [22], [23]. This approach has several benefits: first, it allows for assessment of connectivity rather than activation, and second, it does not require a potentially confounding task. A self-report inventory that independently measures positive and negative affect, such as the Positive and Negative Affect Schedule (PANAS), allows for examination of all four hypotheses by correlating the individual scores of the questionnaire's scales to the strength of connectivity between brain regions. If the first hypothesis were correct, we would expect that the networks for both positive affect (PA) and negative affect (NA) are found primarily in the right hemisphere. If the second view were correct, we would expect that networks for PA are primarily located in the left hemisphere, whereas networks for NA are found primarily in the right hemisphere. For the third view, we would assume that networks for positive and negative affect substantially overlap. For the fourth view, we would expect to see different brain regions for PA and NA, e.g., connectivity of basal ganglia being correlated to PA and connectivity of amygdala, insula, subgenual anterior cingulate being correlated to NA. We tested these hypotheses by investigating how whole-brain functional connectivity from 200 regions-of-interest covering the gray matter [24] covaried with PANAS scores for PA and NA across 65 participants. Our results showed a more complex pattern than that hypothesized by each theory alone. In line with mixed findings throughout the relevant literature, we found a joint network for positive and negative affect, but also uncovered localized elements specific to each affect with a general lateralization trend to the right being present.

## Methods

### Ethics Statement

Approval from the local ethics boards of the Max Planck Institute in Leipzig and the Charité Hospital in Berlin and written informed consent of the participants was obtained.

### Participants

Out of 81 healthy adults who participated in four different research projects at the Max Planck Institute in Leipzig and the Charité Hospital in Berlin (see Table 1 for details) and had data collected for the purpose of this study, 65 were used for analysis. Notably, the resting-state scan was always performed prior to any other task. Two were excluded as their PANAS scores indicated anxiety (NA>29), as suggested by Crawford and Henry [25] and their scores exceeded 2.5 SD from the group mean; another two participants were excluded for exceeding 2.5 SD from the group mean in age. Further, eight participants were excluded due to excessive head motion in the fMRI data. Levene's test was then used to establish equality of variances in PANAS scores, age and sex across the four participant groups (*p>*0.17, *n.s.*) before employing a one-way ANOVA to ensure the groups were not significantly different in either of these domains, leading to the exclusion of another four participants. In the end, 32 females were among the 65 participants (mean age 27.6, SD 3.6; group sizes n = 28, n = 10, n = 16 and n = 11). All participants were right-handed and had no psychiatric or neurological history. The data is available for download at: http://fcon_1000.projects.nitrc.org/.

**Table 1:** Participant details and acquisition parameters.

Voxel size (mm^3^)	3×3×4 (gap: 1 mm)	3×3×4 (gap: 1 mm)	3×3×4 (gap: 1 mm)	3.44×3.44×3.6 (gap: 20%)	*-*
**No. slices**	34	34	34	30	*-*
**Flip angle**	90°	90°	90°	90°	*-*
**TE (ms)**	30	30	30	30	*-*
**TR (ms)**	2300	2300	2300	2008	*-*
**Scanner**	Siemens Magnetom Trio Tim 3T	Siemens Magnetom Trio Tim 3T	Siemens Magnetom Trio Tim 3T	Siemens Verio 3T	*-*
**No. volumes**	200	200	200	240	*-*
**NA Mean (sd)**	18.93 (4.45)	18.7 (5.03)	18 (3.5)	19.91 (3.94)	*18.69 (4.28)*
**PA Mean (sd)**	34.04 (6.22)	35 (3.68)	35.81 (3.73)	33.64 (5.78)	*34.51 (5.24)*
**Time-frame**	in general	in general	in general	last twelve months	*-*
**Schedule Version**	PANAS	PANAS	PANAS	PANAS-X	*-*
**Age Mean (sd)**	28.65 (3.59)	25.32 (3.11)	27.13 (3.19)	27.52 (3.85)	*27.57 (3.6)*
**Participants (Females)**	28 (13)	10 (6)	16 (7)	11 (7)	*65 (33)*
	Berlin	Leipzig 1	Leipzig 2	Leipzig 3	*Total*

### Positive and Negative Affect Schedule

**Figure 2:**
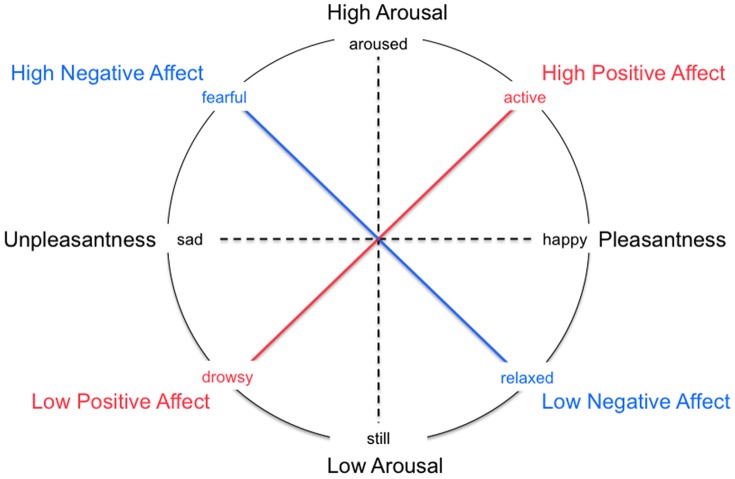
The PANAS captures two independent dimensions of Positive Affect (PA) and Negative Affect (NA). (Figure based on [32]). In this circumplex model of affective space, they fall between the Pleasantness and Arousal spectra, two dimensions which are orthogonal themselves. The PANAS scales emerge after rotation of these two factors. The correlation between the PA and NA scales is low enough to suggest relative independence when taking the measurement error into account.

The affective tendencies of the participants were assessed with the Positive and Negative Affect Schedule (PANAS), a self-report form designed by Watson, Clark and Tellegen [26] and translated into German by Krohne and colleagues [27]. The PANAS measures positive and negative affective trait and state, and possesses strong reliability and validity [25], [28], [29]. It consists of 20 items, which are self-rated on a 5-point scale: “very slightly or not at all”, “a little”, “moderately”, “quite a bit”, and “extremely”. Examples for positive affect (PA) include “active” or “inspired”, and for negative affect (NA) “scared” or “ashamed” (see Table 2 for list of items). At one site, we collected the PANAS-X, a 60-item extended version of the PANAS, and considered the 20 items that were consistent with the original version. A high level of PA is described as “a state of energy, full concentration and pleasurable engagement”; a low level of PA is “characterized by sadness and lethargy”; a high level of NA “subsumes a variety of aversive mood states, including anger, contempt, disgust, guilt, fear, and nervousness”; and a low level of NA is regarded as “a state of calmness and serenity” [30]. Therefore, PANAS PA and NA do not represent opposite ends of the Pleasantness-Unpleasantness spectrum as PA and NA of other affective scales do. Rather, they are designed to be orthogonal and independent of each other. In a prominent depiction of affective space, called the circumplex model [31], the PA and NA scales fall between the pleasantness and arousal spectra, two dimensions which are orthogonal themselves. The PANAS scales emerge after rotation of these two factors (i.e., pleasantness and arousal) [32], [33] (see Figure 2). The correlation between the PA and NA scales has been shown to be low enough to suggest relative independence when taking the measurement error into account [30]. The PANAS allows for the flexibility of addressing both state and trait affect to varying degrees depending on the timeframe participants are asked to consider when responding (e.g. “right now”, “during the last week”, “during the last year”) [26]. In the current study, participants were asked to consider the items “in general” or “the last twelve months”, in order to provide scores of affective disposition (rather than state specific responses).

**Table 2:** Positive and Negative Schedule (PANAS) items.

Positive Affect (PA)	Negative Affect (NA)
Interested	Distressed
Excited	Upset
Strong	Guilty
Enthusiastic	Scared
Proud	Hostile
Alert	Irritable
Inspired	Ashamed
Determined	Nervous
Attentive	Jittery
Active	Afraid

### fMRI Data Acquisition

Resting-state fMRI data was collected on Siemens Magnetom Tim Trio scanners at both sites, and a Siemens Verio 3 Tesla scanner in Leipzig. Imaging protocols varied slightly between datasets (for complete list of parameters refer to Table 1). Only fMRI datasets with less than 1 mm of maximum head motion in any dimension were included in the analysis; eight subjects were excluded due to this criterion.

### Data Analysis

#### fMRI Data Preprocessing

**Figure 3:**
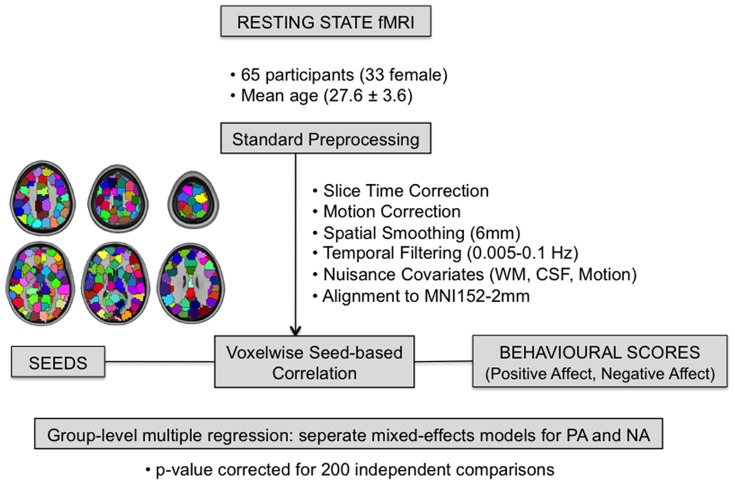
Data processing path. Following standard pre-processing of the resting state fMRI data, connectivity was calculated between the time courses of each of the 200 functional seeds and all the voxels in the brain. These connectivity scores were correlated with PA and NA scores in separate analyses, which were followed by a group-level multiple regression and two conjunction analyses.

fMRI data was preprocessed based on modified versions of the 1000 functional connectome scripts [34], available at: www.nitrc.org/projects/fcon_1000. The scripts use tools from both AFNI [35] and FSL [36]. The procedure consisted of slice-time correction, motion correction and spatial smoothing (6 mm Gaussian kernel), as well as temporal band-pass filtering between 0.005–0.1 Hz, removal of linear and quadratic trends, and linear registration to 2×2×2 mm MNI152 standard space. ‘Nuisance’ signals, including white matter, cerebral spinal fluid and the six motion parameters, were removed from the data using a multiple regression (see Figure 3 for processing path). We did not remove the global signal [37], [38].

### Seed-based functional connectivity analysis

In order to investigate how functional connectivity across the whole-brain co-varies with affective disposition scores, we correlated the individual scores in the PA and NA scales of the PANAS with connectivity values derived from a seed-based functional connectivity analysis. The seed-based connectivity analysis was based on 200 parcellation units created by Craddock and colleagues [24] as seed regions (see Figure 3). In contrast to parcellations such as the Harvard-Oxford and AAL masks, these units are based on functional connectivity rather than anatomy, and are similar in size. The average time-course within each parcellation unit was extracted, and then correlated with the time-course of every other voxel in the brain. Resultant whole-brain correlation maps were normalized using Fisher's *r*-to-*z* transform (z = [ln(1+r)−ln(1-r)]/2) for comparison across individuals.

### Group-level analysis

Group-level statistical tests were performed using FSL's least squared mixed-effects analysis FEAT [36]. De-meaned PA and NA scores were included as covariates of interest and analyzed in separate models to avoid a priori influences of the respective dimensions on each other. De-meaned scores for age, sex and site were included as covariates of no interest. Therefore, each model examined how much PA or NA, age, sex and site predict the strength of connectivity between each of the seeds and all other voxels in the brain. Significance was assessed using a z>2.3 voxel-wise threshold, and cluster correction using Gaussian Random Field theory with p<0.05. The p-value was additionally Bonferroni-corrected for the 200 independent ROIs (p<0.00025).

To examine common functional connectivity across both PA and NA, two conjunction analyses were carried out. The first examined overlapping voxels across functional connectivity from all ROIs; the second examined every ROI individually to investigate if PA and NA shared any of the same connections.

### Lateralization Index (LI)

In order to test for laterality preference, the significant voxels were counted separately per hemisphere for every connection between cluster and seed ROI. If clusters or seeds were distributed across the hemispheres, voxels within the cluster or seed were also calculated separately for each hemisphere. Where clusters overlapped, voxels within the overlap were counted for each significant cluster. The lateralization indexes were then calculated using the formula LI = (QLH-QRH)/(QLH+QRH), LH and RH indicating the left and right hemisphere, and Q meaning the quantity of voxel surpassing threshold. Laterality was then assessed using a standard LI threshold of 0.2 (LITH, equaling 50% more voxels in one hemisphere than the other) and the following rule: LI>LITH = left hemispheric dominance; LI < - LITH = right hemispheric dominance, and |LI|≤LITH = no hemispheric dominance [39]. As the LI values can vary depending on the statistical threshold used, we computed the LI at the additional thresholds of p<0.0005 and p<0.001.

## Results

### Positive and Negative Affect Scores

**Figure 4:**
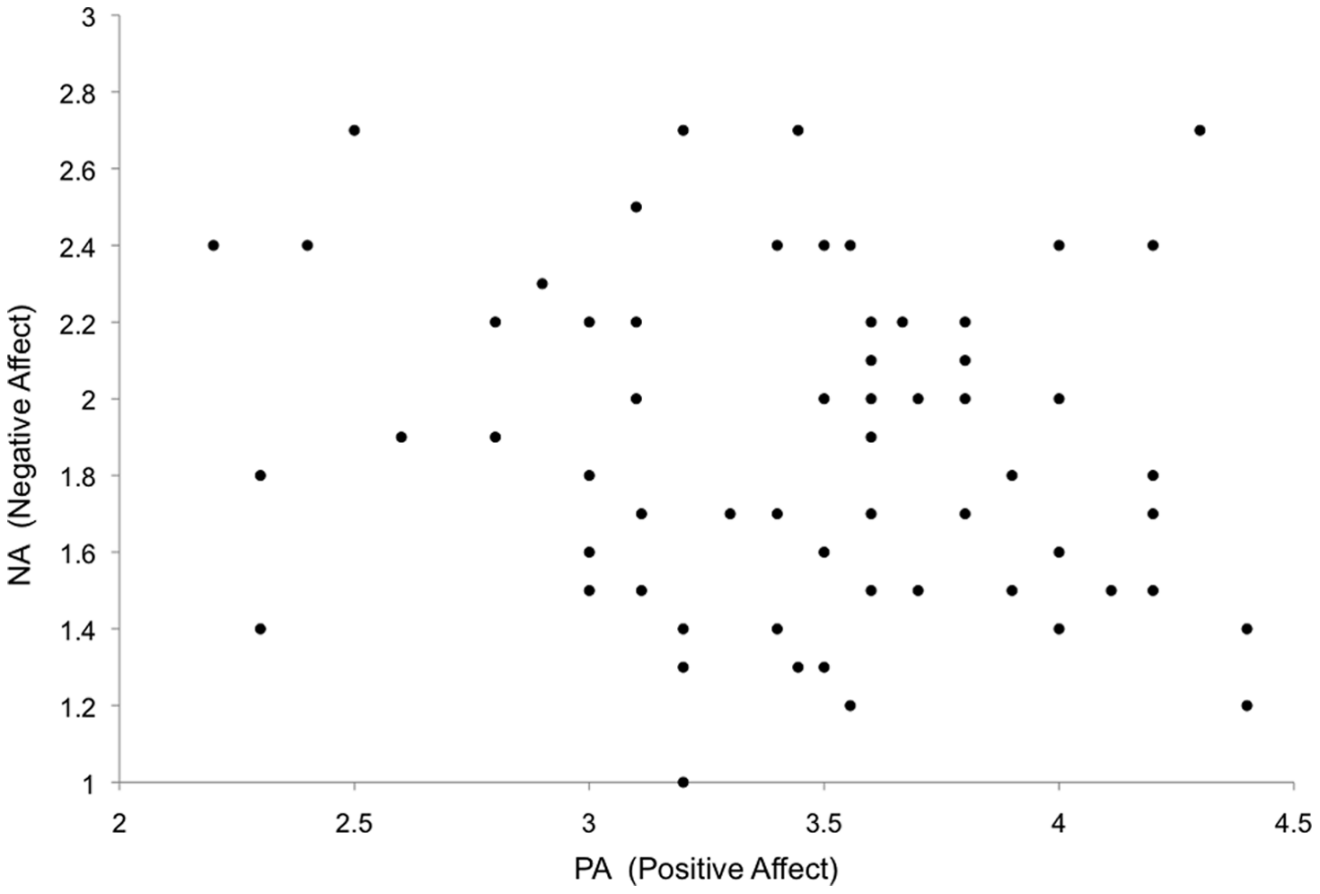
No correlation between the scales. A correlation analysis revealed no correlation between individual scores of NA and PA (r^2^ = 0.025, p>0.4), as expected from their construction as orthogonal scales (see Figure 2).

Average participant scores on PA (mean 34.6 ± SD 5.4; range 22–44) and NA (mean 18.7 ± SD 4.3; range 10–27) resembled those of prior studies conducted with healthy individuals in the trait domain ([27]: PA: mean 32.9, NA: mean 18.4). Furthermore, there was no correlation between individual scores of PA and NA (r^2^ = 0.025, p>0.4; see Figure 4), as expected based on their construction as independent dimensions.

### Lateralization

At an LI of −0.293, NA showed a right-sided dominance, while PA emerged bilaterally dominant at −0.15. Using the standard LITH of 0.2, neither classification changed as the statistical threshold was lowered: at p<0.0005, the LIs for PA and NA were −0.085 and −0.228 and at p<0.001 they were −0.058 and −0.241, respectively. LI values for all individual connections can be found in Tables 3 and 4, as well as in Tables S1, S2, S3 and S4 for the additional thresholds.

**Table 3:** Details of connections for NA.

	Correlation with NA	Lat	Seed ROI	Lat	Connectivity Cluster	Cluster Size (mm^3^)	Cluster p-value	Peak Z value	x	y	z	Voxels LH in %	Voxels RH in %	LI	Domi-nance
1	positive	R	OP, LG	R	SG, S1, SPL	1890	3.7×10^−5^	4.04	52	−26	36	0	100	−1	R
2	positive	L	dlPFC	R	SG, SPL, OL, PCN	1777	9.06×10^−6^	4.27	40	−44	54	0	100	−1	R
3	positive	R	LG, TF, OF	R	SG, SPL, OL	1509	0.00023	4.24	36	−34	30	0	100	−1	R
4	positive	L	LG, TF, OF	R	SG, SPL, OL	1850	3.73×10^−5^	3.92	40	−46	56	33.76	66.24	−0.32	R
5	positive	R	SPL	BIL	CN, LG, OP, OL	6945	7.56×10^−10^	4.13	−14	−74	−18	51.79	48.21	0.04	BIL
6	positive	R	LG, CALC	R	SG, S1	1545	0.000217	4.39	50	−28	36	34.42	65.58	−0.31	R
7	positive	L	SPL	BIL	CN, LG, OP	4924	4.17×10^−7^	3.94	16	−74	26	45.4	54.6	−0.09	BIL
8	negaitve	BIL	SMA	BIL	adACC, pgACC, rmPFC	1879	3.86×10^−5^	4.47	6	18	32	42.4	57.8	−0.16	BIL
9	negaitve	BIL	Ventral Striatum	BIL	M1, S1, SMA	3194	1.97×10^−6^	3.93	−34	−26	38	50.63	49.37	0.01	BIL
10	negaitve	R	CRBL, Pons	BIL	CN, OL, OP	2264	0.000167	3.86	12	−80	36	47.45	52.55	−0.05	BIL

adACC = anterior dorsal anterior cingulate, CALC = Intracalcarine Cortex, CN = Cuneal Cortex, CRBL = Cerebellum, dlPFC = dorsolateral prefrontal cortex, LG = Lingual Gyrus, M1 = Primary Motor Cortex, OF = Occipital Fusiform, OL = Lateral Occipital Complex, OP =  Occipital Pole, PCN = Precuneus, pgACC = perigenual anterior cingulate , rmPFC = rostromedial prefrontal cortex, S1 = Primary Somatosensory Cortex, SG = Supramarginal Gyrus, SMA = Supplementary Motor Area, SPL = superior parietal lobule, TF = Temporal Fusiform, TP = temporal pole.

**Table 4:** Details of connections for PA.

	Correlation with PA	Lat	Seed ROI	Lat	Connectivity Cluster	Cluster Size (mm^3^)	Cluster p-value	Peak Z value	x	y	z	Voxels LH in %	Voxels RH in %	LI	Domi-nance
1	negaitve	R	CRBL	BIL	LG, CALC, OP, OL, TF, OF	2335	4.04×10^−5^	3.8	−2	−90	−8	22.14	77.86	−0.56	R
2	negaitve	L	CRBL	BIL	SMA, PMC, SG, M1, S1, PCC, pdACC. dlPFC	3349	1.07×10^−5^	4.08	30	−10	64	31.71	68.29	−0.37	R
3	negaitve	L	Posterior Medial Temporal	R	Ins, STS, Put, M1, S1, S2, dlPFC	1978	0.000227	4.24	42	−20	14	31.82	68.18	−0.36	R
4	negaitve	L	CRBL, Pons	R	STG, TP, Ins, M1, S1	2671	1.72×10^−5^	4.4	50	−10	−4	18.23	81.77	−0.64	R
5	negaitve	R	TP	BIL	CRBL, Pons	2589	4.32×10^−5^	4.34	−14	−34	−28	41.9	58.1	−0.16	BIL
6	negaitve	L	CRBL	BIL	OP, OL, LG, CALC, CRBL, PCN	8365	8.25×10^−10^	4.93	8	−90	40	56.45	43.55	0.13	BIL
7	negaitve	L	Thalamus	BIL	sgACC, pgACC, adACC, rmPFC, dmPFC, FP	3079	2.62×10^−5^	4.78	−12	26	28	48.17	51.83	−0.04	BIL
8	negaitve	R	dlPFC	BIL	OP, LG, Vermis, TF, OF	2041	1.74×10^−5^	3.61	−8	−72	14	31.35	68.65	−0.37	R
9	negaitve	R	Caudate	BIL	1. PCC, PCN, pHip, mOFC, dlPFC	1. 3478	3.28×10^−6^	4.28	−4	−36	28	58.62	41.38	0.17	BIL
					2. FP, sgACC, pgACC, vmPFC, rmPFC,	2. 2821	2.71×10^−5^	4.21	−16	34	−16				

adACC = anterior dorsal anterior cingulate, CALC = Intracalcarine Cortex, CN = Cuneal Cortex, CRBL = Cerebellum, dlPFC = dorsolateral prefrontal cortex, dmPFC = dorsomedial prefrontal cortex, FP = frontal pole, Ins = Insula, LG = Lingual Gyrus, M1 = Primary Motor Cortex, mOFC = medial OFC, OF = Occipital Fusiform, OL = Lateral Occipital Complex, OP =  Occipital Pole, PCC = posterior cingulate, PCN = Precuneus, pdACC = posterior dorsal anterior cingulate, pgACC = perigenual anterior cingulate , pHip = posterior Hippocampus, PMC = Premotor Cortex, Put = Somatosensory Cortex, SG = Supramarginal Gyrus, sgACC = subgenual anterior cingulate, SMA = Supplementary Motor Area, STG = superior temporal gyrus, STS = superior temporal sulcus, TF = Temporal Fusiform, TP = temporal pole, vmPFC = ventromedial prefrontal cortexPutamen, rmPFC = rostromedial prefrontal cortex, S1 = Primary Somatosensory Cortex, S2 = Secondary.

### Affective disposition reflected in functional connectivity patterns

**Figure 5:**
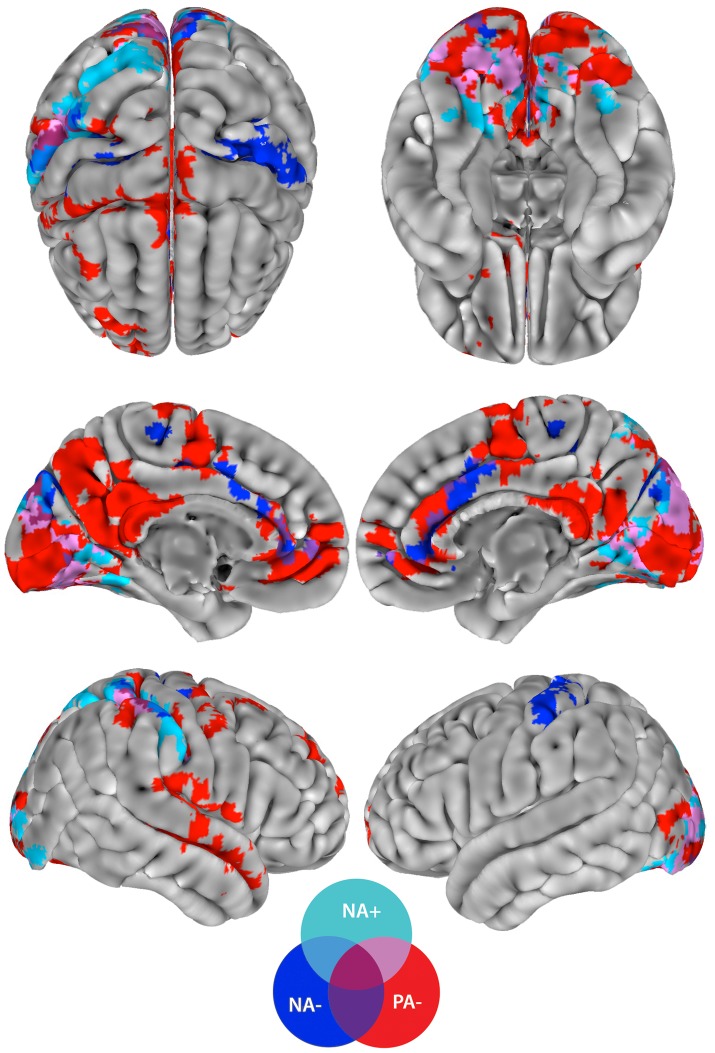
Networks correlated with Positive Affect (PA) and Negative Affect (NA). Whole-brain connectivity analysis revealed networks that were correlated with PA or NA. While some of the regions were common to both PA and NA functional connectivity patterns, others were dissociative of the respective affective domain, here depicted in different colors. A trend for overall right-hemispheric dominance was observed.

Whole-brain connectivity analysis revealed networks that were correlated with either PA or NA (see Tables 3 and 4, and Figure 5; see also Figures S4, S5, S6, S7). These networks included regions previously implicated in reactions to emotional stimuli, such as the anterior cingulate and the insula. While for example the anterior cingulate was common to both PA and NA functional connectivity patterns, the insula and other regions were dissociative of the respective affective domain, as will be detailed below.

### Common brain areas, but not connections, across positive and negative affect

A first conjunction analysis across all significant results (independent of specific ROIs) revealed regions that were significantly related to both PA and NA. These areas included the medial prefrontal cortex (mPFC), anterior cingulate (ACC), visual cortex, cerebellum, supplementary motor area (SMA), supramarginal gyrus and somatosensory cortex (see Figure 5 and Figures S4, S5, S6, S7). An independent second conjunction analysis that investigated the connections from every ROI to all voxels did not yield any common results across PA and NA. For example, PA was correlated with the mPFC's connectivity with the cerebellum, the thalamus and the caudate, but NA was correlated with the mPFC's connectivity with the SMA. Despite some regions being shared by PA and NA networks, none of the connections within these networks were identical.

### Dissociative connectivity patterns for positive and negative affect

Our separate analysis models for each domain found three networks: a network that was positively correlated with NA, a network that was negatively correlated with NA, and a network that was negatively correlated with PA. Within the positively correlated network, greater connectivity was observed with higher NA, whereas within the negatively correlated networks, greater connectivity was observed with either lower NA or PA.

**Figure 6:**
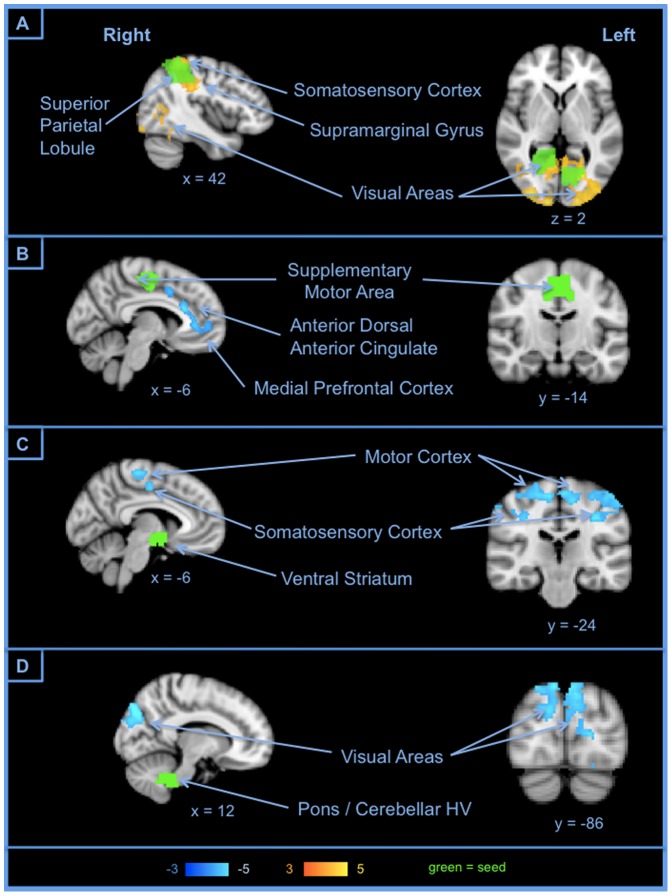
Examples of connections correlated with Negative Affect. NA was reflected in two small networks: within the positively correlated network, greater connectivity was observed with higher NA, whereas within the negatively correlated network greater connectivity was observed with lower NA.

The network positively correlated with NA included several regions, which were densely connected and consisted mainly of reciprocal connectivity between visual areas and supramarginal gyrus, superior parietal lobule (SPL) and somatosensory cortex (Figure 6A). NA's negatively correlated network was more spatially dispersed: the SMA connected to mPFC and the dorsal anterior cingulate (dACC) (Figure 6B); the ventral striatum connected to primary somatosensory and motor cortex (Figure 6C); and the left cerebellar segment HV and the pons of the brainstem connected to visual areas (Figure 6D).

**Figure 7:**
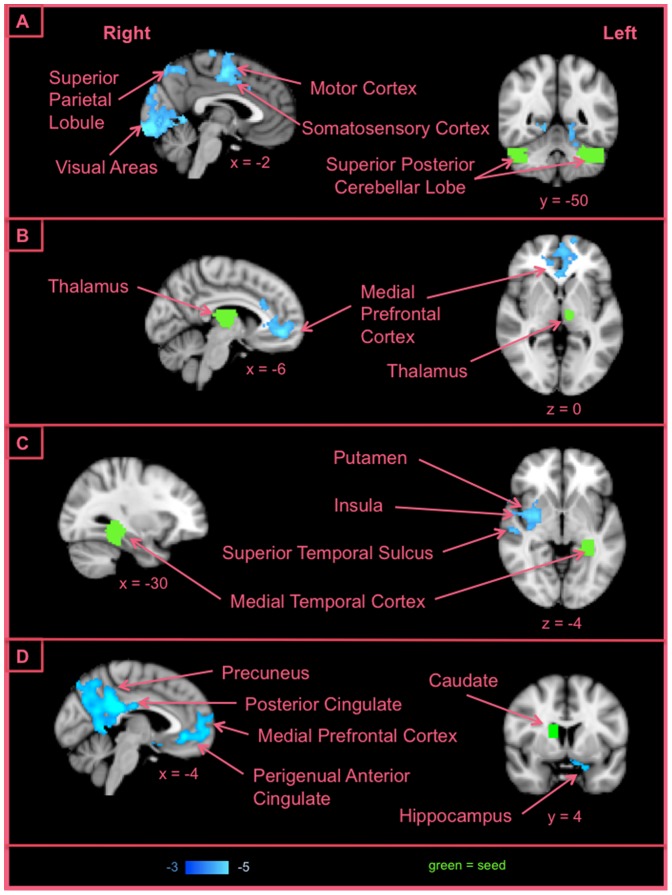
Examples of connections correlated with Positive Affect. We detected a large network that was negatively correlated to the PA score; greater connectivity within this network was observed to correlate with lower PA.

For PA, we detected a large network that was negatively correlated to the score; greater connectivity within this network was observed to correlate with lower PA. Key components were connections between bilateral seeds in the superior posterior lobe of the cerebellum and bilateral visual, superior parietal and sensorimotor areas (Figure 7A). This analysis further revealed connectivity between the thalamus and the mPFC (Figure 7B); connectivity between the left posterior medial temporal cortex and right insula, superior temporal sulcus (STS) and putamen (Figure 7C) and connectivity between the caudate and perigenual anterior cingulate (pgACC)/ventromedial prefrontal cortex (vmPFC), as well as to posterior cingulate (PCC)/precuneus and the hippocampus (Figure 7D).

## Discussion

We tested four hypotheses on affective organization in the brain by using resting-state functional connectivity data and scores from the Positive And Negative Affective Schedule (PANAS). The first suggests a right-lateralized dominance in affective processing, the second a hemispheric dominance according to positive or negative valence, the third one-network for all affective processing and the fourth a localized processing of discrete emotions. In the lateralization analyses, a trend for NA being processed more in the right hemisphere than in the left emerged. Conjunction analyses revealed regions that are involved in both affective dimensions, in line with the network/constructionist hypothesis. The respective connections of these regions differed, however, between PA and NA, pointing to the presence of localized elements. These results furthermore support the potential for using resting state fMRI to investigate the relationship between brain organization and behavioral phenotypes.

### Lateralization of affective disposition

To examine the lateralization, which is at the core of the first two hypotheses, we calculated the significant voxels in each hemisphere separately for comparison and used the Lateralization Index (LI) to assess laterality. When estimating the LI scores for every significant cluster separately, NA showed a right-sided dominance effect, while PA appeared to be bilaterally dominant regardless of the specific statistical threshold used. The right hemisphere is thought to contain essential components of systems needed in emotion processing, as it has been found to be more active than the left hemisphere in both the processing of visual [1] and auditory [2] emotional stimuli. In these studies, performance of the hemispheres was compared directly by presenting the affective input to only one eye or ear. Moreover, lesions in the right hemisphere have been shown to impact the recognition of emotional faces, whereas this was not the case for left-hemispheric lesions, even though lesion size and site was matched [3]. Aside from affective processing, hemispheric specialization has also been observed e.g. in language [40] and spatial attention [41]–[43]. It has been suggested that lateralization allows for more efficient parallel processing, decreases redundancy of neural operations, and increases the computational speed of cognitive processes [44]. Our data here provides partial support for both hypotheses, as it suggests the right hemisphere being more dominant in negative affective processing while neither showing a general affective lateralization towards the right nor a left-hemispheric dominance in positive affect.

### Regions of affective disposition

Regions that were associated with both PA and NA were mPFC, ACC, SMA, somatosensory cortex, supramarginal gyrus, visual cortex and cerebellum.

The mPFC was found in numerous studies of emotion and is commonly active during emotional stimulation, irrespective of any specific emotion or stimulation method [45], [46]. One potential explanation is that the mPFC may have a role in detecting emotional signals, regardless of whether they are internally or externally generated [47]. It has been implicated in theory of mind and the social cognition aspect of it [48], [49], as it has been found active e.g. during self-referential mental activity [50], observing social interactions [51], and understanding psychological aspects about others [52]. Another account is that mPFC may be involved in the allocation of cognitive resources to modulate emotional arousal in a regulatory effort [53]. The ventral and rostral portions of the ACC are typically thought of as centers of emotion control. For example, activation in this region was higher for people who took longer to evaluate negative vs. neutral information that was presented in a stimulus [54], and it was also enhanced in depressed patients in a stop signal task as compared to healthy controls [55]. In contrast, dACC (in particular its anterior portion) has been frequently implicated in conflict monitoring and management in more purely cognitive studies [56]–[58]. In addition, it was suggested that the dACC impacts the amygdala via its connections with the most ventral part of the ACC, the subgenual ACC (sgACC) [58]. The SMA is thought to connect the ACC and mPFC to motor areas as well as to another region of cognitive control, namely the dlPFC [59].

Consistent with the idea that we understand emotional states by internally generated somatosensory representations, the somatosensory cortex has been associated with the recognition of emotional expression [60], [61]. The supramarginal gyrus has been implicated in emotional judgments [62], the reappraisal of emotional stimuli [63], as well as social perception and empathy [64]. While supramarginal gyrus was also involved in both affective dispositions, it was much more prominent in negative affect (see next section). Activation of visual areas in studies of emotion was typically thought to be due to the visual paradigms; however, visual areas are also active in auditory [65] and olfactory emotional stimulation [66] and show up when normal [22] and pathological personality traits [67], [68] are investigated, suggesting them to be implicated in the conjuring up of emotional mental images. Much in line with our findings, the study of Adelstein et al. [22] which used the NEO Five-Factor Inventory [69] to explore the brain's resting-state connectivity underlying personality traits, the visual cortex exhibited differential connectivity that was correlated to all five personality domains. Liao and colleagues found an enhanced effective connectivity between amygdala and the visual cortex in patients suffering from social anxiety disorder [68] and Zeng and colleagues detected changes in connectivity from visual cortex that correlated to the presence of major depression [67].

While previously regarded as ‘only a motor region’, the cerebellum has been increasingly implicated in higher cognitive functions [70] as well as emotions. Specifically, it has been postulated to be involved in regulating fear and pleasure responses; the cerebellar vermis has been shown to be involved in four domains of the NEO Five-Factor Inventory in the connectivity study by Adelstein and colleagues [22] with the fifth domain involving the left cerebellar hemisphere; furthermore, changes in cerebellar connectivity were also predictive of major depression [67], and cerebellar lesions have been shown to result in various affective syndromes [71].

Therefore, both PA and NA largely rely on connectivity patterns within a network that is involved in sensory representation and recognition, the regulation and cognitive control of emotion, the resulting motor responses, as well as self-related cognition, potentially within a social context. These findings provide evidence that a number of brain areas are involved in processing many or all emotions, a finding that would be consistent with the network/constructionist hypothesis.

### Distinct functional patterns

While the regions described above were common to both affective domains, they differed with respect to their connectivity, and no connections common to both PA and NA were found. Of note, this finding would not have been yielded by an activation study and may account for some of the discrepancies between the localist and the network/constructionist hypotheses. For example, while the connectivity of anterior cingulate/mPFC with the nearby SMA was negatively correlated with NA, its connectivity with the cerebellum, the thalamus and the caudate negatively correlated with PA. Increased medial prefrontal/anterior cingulate connectivity with the SMA has e.g. been implicated in better control in a Stop signal-task [72]; one could therefore hypothesize that control decreases with higher NA scores. A disruption in the dopaminergic pathway in striatal structures such as the caudate is thought to be involved in the development of depressive symptoms [73], and in line with our findings, an increase in connectivity between cingulate structures and the caudate has been observed in patients suffering from obsessive-compulsive disorder [74], possibly in an effort to increase positive feelings. As the thalamus is typically viewed as the gateway to the cerebral cortex, and thought to regulate conscious state and alertness [75], low positive affect “lethargy” states may also be related to thalamic modulation of the mPFC. Consistent with this idea and our finding that anterior cingulate/medial prefrontal connectivity with the thalamus is negatively correlated with PA, an increase in functional connectivity between the thalamus and anterior cingulate has been shown in depressed patients in comparison to healthy controls [76].

PA and NA networks were not only functionally distinct from each other, but also differed in other aspects. While the network negatively correlated with PA was larger and had numerous connections to subcortical areas, the network positively correlated with NA was smaller and consisted primarily of reciprocal connectivity between visual areas and supramarginal gyrus. Supramarginal gyrus and visual areas might work together to visually identify negative events, judge and reappraise them. Therefore our results also highlight the unique characteristics of the PA and NA networks.

In summary, our data supports certain aspects of several of the proposed theories, which is not entirely surprising as they are not mutually exclusive. Our data is consistent with the views that affective processing may be organized in largely overlapping networks which partly recruit regions that are characteristic for certain emotions as necessary, and preferentially so in the right hemisphere for NA.

### Limitations

While the PANAS, as administered here, is a highly reliable measure of positive and negative affective traits, the generalizability of the current findings is limited to the descriptive scope of this measure. Not all emotion-related networks may be captured by the PANAS, and other methods of evaluating affective differences across individuals such as questionnaires to assess mood, anxiety or depression levels, or performance differences taken from relevant tasks may also provide valuable insights into the relationship between brain organization and affective processing, and could be the basis for future studies.

We have chosen to use an exploratory ROI based approach in order to query whole-brain connectivity related to variance in the positive and negative PANAS scores. Such a method assumes independence of the ROIs from one another, and group-level cluster correction may be overly conservative, resulting in false negatives. This approach, however, was preferable to an a priori selection of specific ROIs, which would have restricted the exploratory nature of the current analysis. Importantly, in our additional statistical thresholds of p<0.0005 and p<0.001, no regions are involved that have not already been detected at p<0.00025. Visually identifiable differences are sparse (see Figures S1 and S2) and additional connections typically replicate already observed patterns (see Tables S1, S2, S3 and S4). While the involvement of the amygdala in a study of emotions would be hypothesized, it remained notably absent from our results. This may be down to the aforementioned limitations of our study, but could also be a negative finding. Several examples for similar cases exist: for instance, the amygdala was not detected in studies on sustained mood states [77], [78]. We therefore performed a complementary hypothesis-driven analysis with only those seeds that contained the amygdala. Differential patterns in functional connectivity of the right amygdala were associated with both PA and NA (Figure S3), although only at low significance (p<0.05).

Further, some differences in population and PANAS version across the four groups of participants remained even after the exclusion of several subjects to ensure the groups were equal in variances and not significantly different in means. However, these should be accounted for alongside the differences in scanner site by an additional covariate in both our models.

Appropriately powered studies are still a concern for investigating phenotypic measures and resting state functional connectivity. Ongoing large-scale data acquisition initiatives are enabling increasingly larger studies to be conducted [34], [79]. As we have included 65 datasets across four different study protocols there is reason to believe that these findings will be replicable. Future studies using independent groups will be valuable for validating the current findings.

## Conclusion

By investigating how whole-brain functional connectivity from 200 regions-of-interest covaried with PANAS scores for positive and negative affect, we tested four previously suggested hypotheses about how affective processing is organized in the brain. Our results provide some support for both theories about lateralization, as they show a right-sided dominance effect for negative affective processing, but while they do not currently suggest a general lateralization of affective processing to be present throughout the brain, they also suggest positive affective processing to be bilaterally dominant. Our data is mostly in line with the hypothesis of a largely overlapping joint network basic to processing of all emotions, as revealed by the conjunction analysis of overlapping regions. Additionally, as shown through different results in connectivity, there is also some support for the hypothesis that processing of certain emotions may recruit distinct regions. However, because of the substantial overlap, it seems unlikely that these should be independent. In essence, our findings suggest a network-based or constructionist framework between localized elements responsible for affective processing, which is more dominant in the right than in the left hemisphere for negative affect.

## Supporting Information

Figure S1 **Networks correlated with PA and NA at a statistical threshold of p<0.0005.** In order to assess the likelihood of false negatives, we lowered our threshold to p<0.0005. Differences (new or newly overlapping voxels) to the originally employed threshold of p<0.00025 are circled in black.
(TIF)

Figure S2 **Networks correlated with PA and NA at a statistical threshold of p<0.001.** In order to further assess the likelihood of false negatives, we lowered our threshold further to p<0.001. Differences (new or newly overlapping voxels) to the threshold of p<0.0005 are circled in black.
(TIF)

Figure S3 **Amygdala connectivity covaries with PA and NA.** At a threshold of p<0.05, a negative correlation was observed between both (A) NA and (B) PA connectivity from the amygdala to differential regions in the brain.
(TIF)

Figure S4 **Axial slices of connections 1 to 5 correlated with NA.** Details of the displayed connections can be found in Table 3.
(TIF)

Figure S5 **Axial slices of connections 6 to 10 correlated with NA.** Details of the displayed connections can be found in Table 3.
(TIF)

Figure S6 **Axial slices of connections 1 to 5 correlated with PA.** Details of the displayed connections can be found in Table 4.
(TIF)

Figure S7 **Axial slices of connections 6 to 9 correlated with PA.** Details of the displayed connections can be found in Table 4.
(TIF)

Table S1 Details of additional connections at p<0.0005 for NA.
(DOC)

Table S2 Details of additional connections at p<0.0005 for PA.
(DOC)

Table S3 Details of additional connections at p<0.001 for NA.
(DOC)

Table S4 Details of additional connections at p<0.001 for PA.
(DOC)
