# Correction: Affect and the Brain's Functional Organization: A Resting-State Connectivity Approach

**DOI:** 10.1371/annotation/a4f9f707-5e23-42f8-9d02-ccdade49750a

**Published:** 2013-12-13

**Authors:** Christiane S. Rohr, Hadas Okon-Singer, R. Cameron Craddock, Arno Villringer, Daniel S. Margulies

Table 1 was published with the incorrect orientation. Please see the corrected Table 1 here: http://plosone.org/corrections/pone.0068015.t001.cn.tif

