# Supplementary figures and images for: Affect and the Brain's Functional Organization: A Resting-State Connectivity Approach
Source: PLoS One. 2013 Jul 23;8(7):e68015. doi: 10.1371/journal.pone.0068015 (PMC3720669; doi:10.1371/journal.pone.0068015)

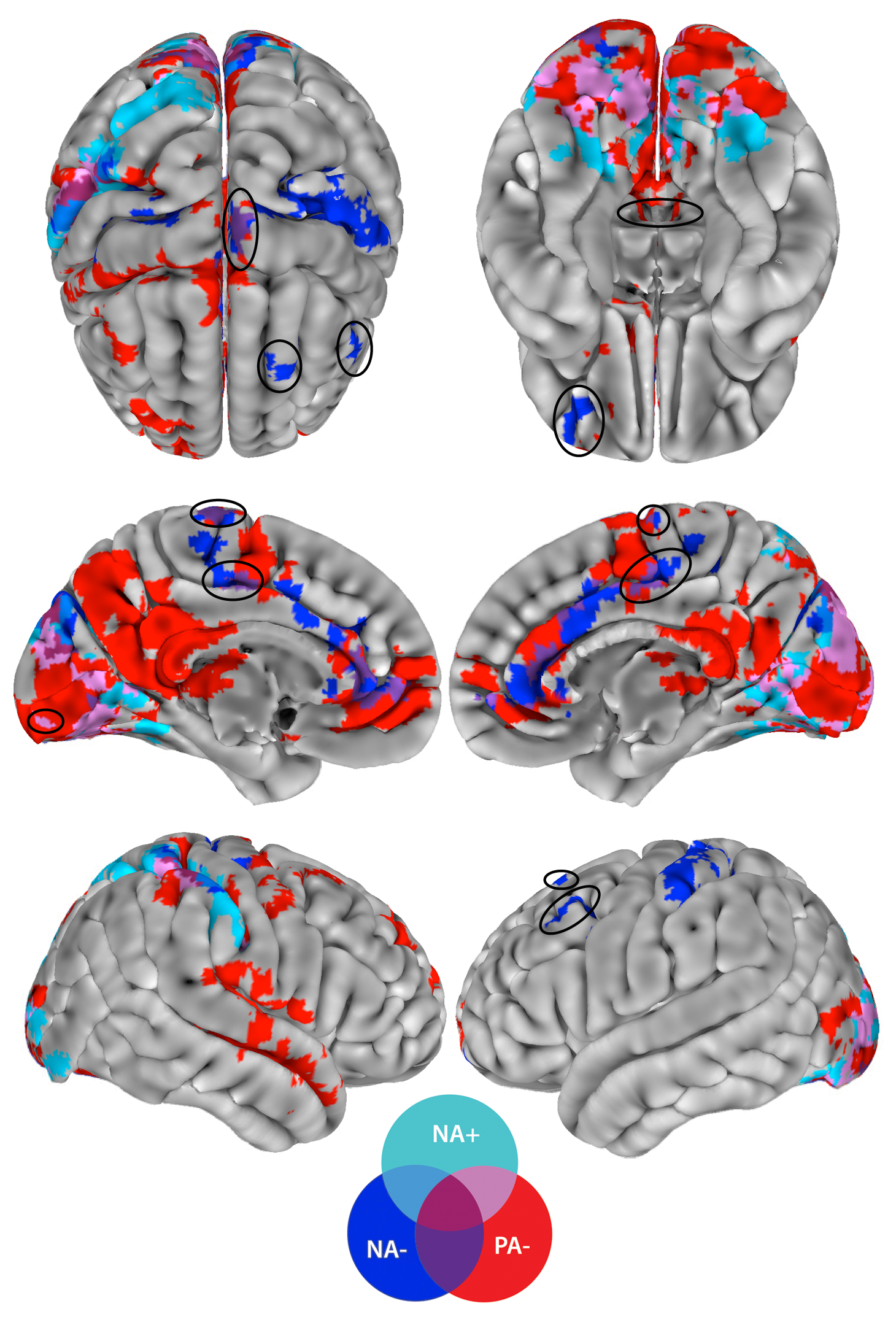

Supplement: Figure S1 — Networks correlated with PA and NA at a statistical threshold of p<0.0005. In order to assess the likelihood of false negatives, we lowered our threshold to p<0.0005. Differences (new or newly overlapping voxels) to the originally employed threshold of p<0.00025 are circled in black. (TIF) [file pone.0068015.s001.tif]

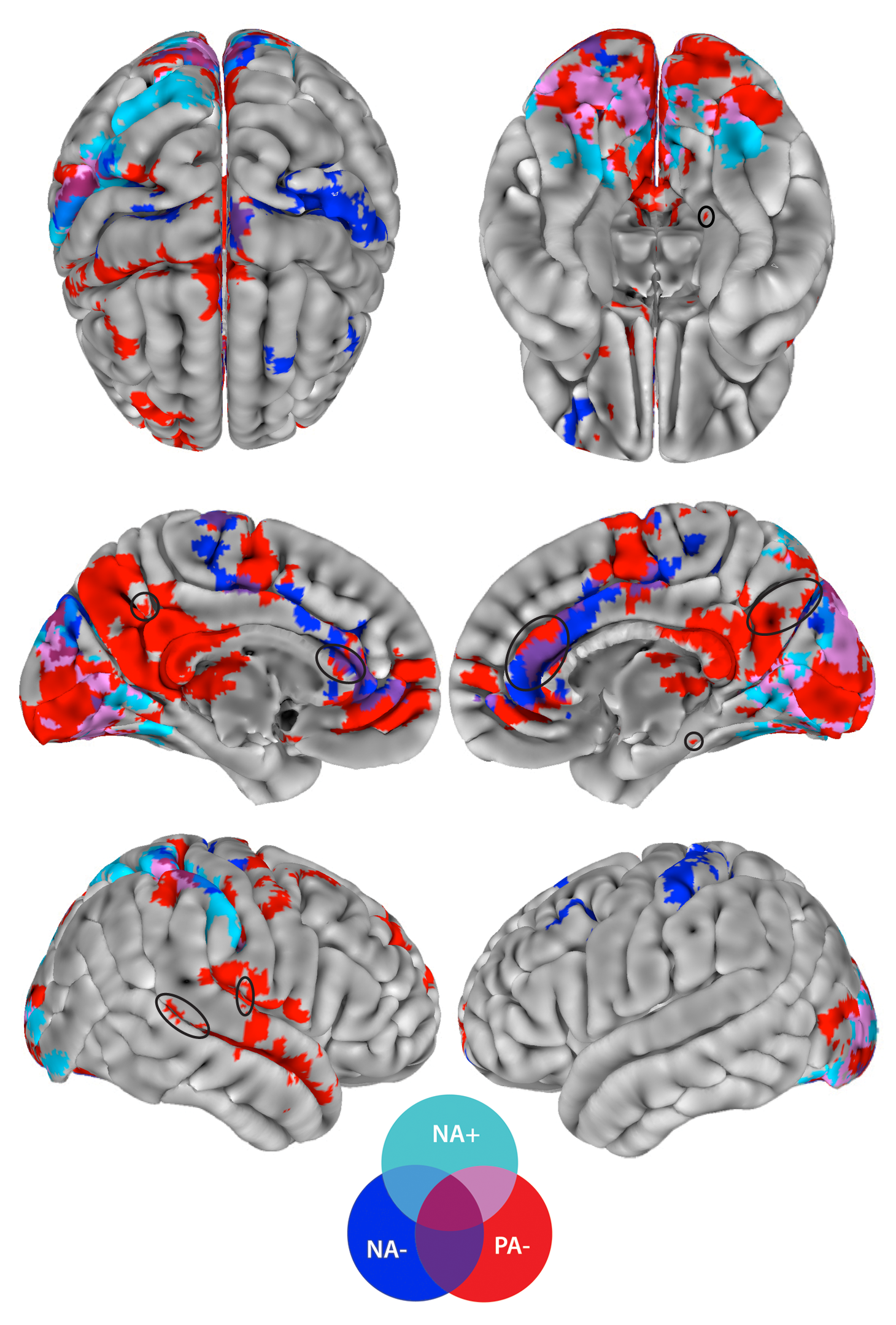

Supplement: Figure S2 — Networks correlated with PA and NA at a statistical threshold of p<0.001. In order to further assess the likelihood of false negatives, we lowered our threshold further to p<0.001. Differences (new or newly overlapping voxels) to the threshold of p<0.0005 are circled in black. (TIF) [file pone.0068015.s002.tif]

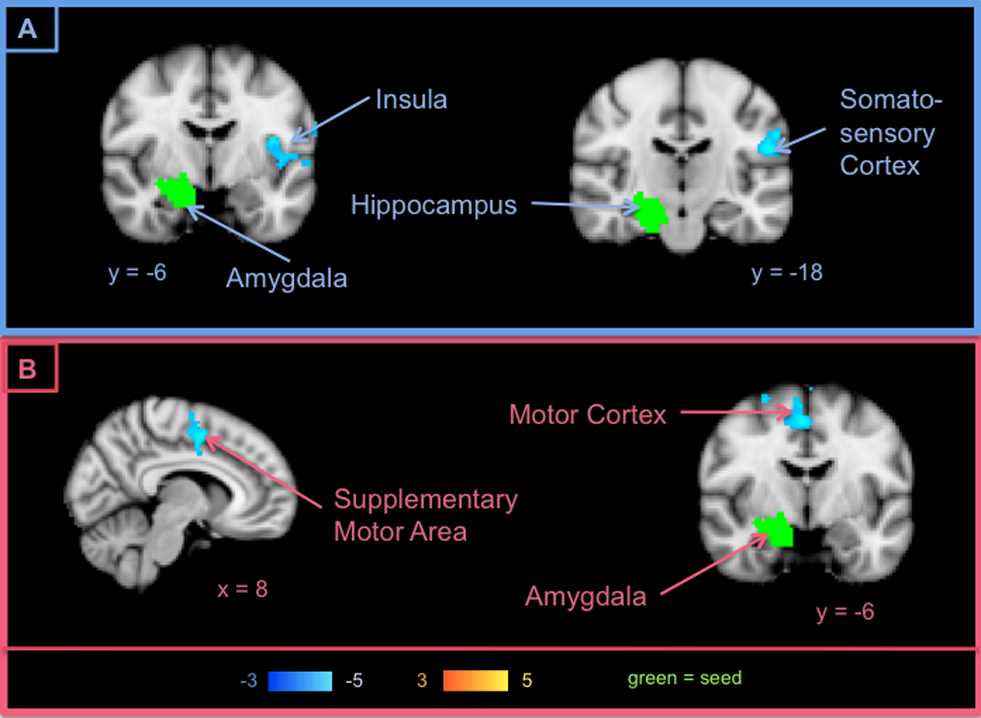

Supplement: Figure S3 — Amygdala connectivity covaries with PA and NA. At a threshold of p<0.05, a negative correlation was observed between both (A) NA and (B) PA connectivity from the amygdala to differential regions in the brain. (TIF) [file pone.0068015.s003.tif]

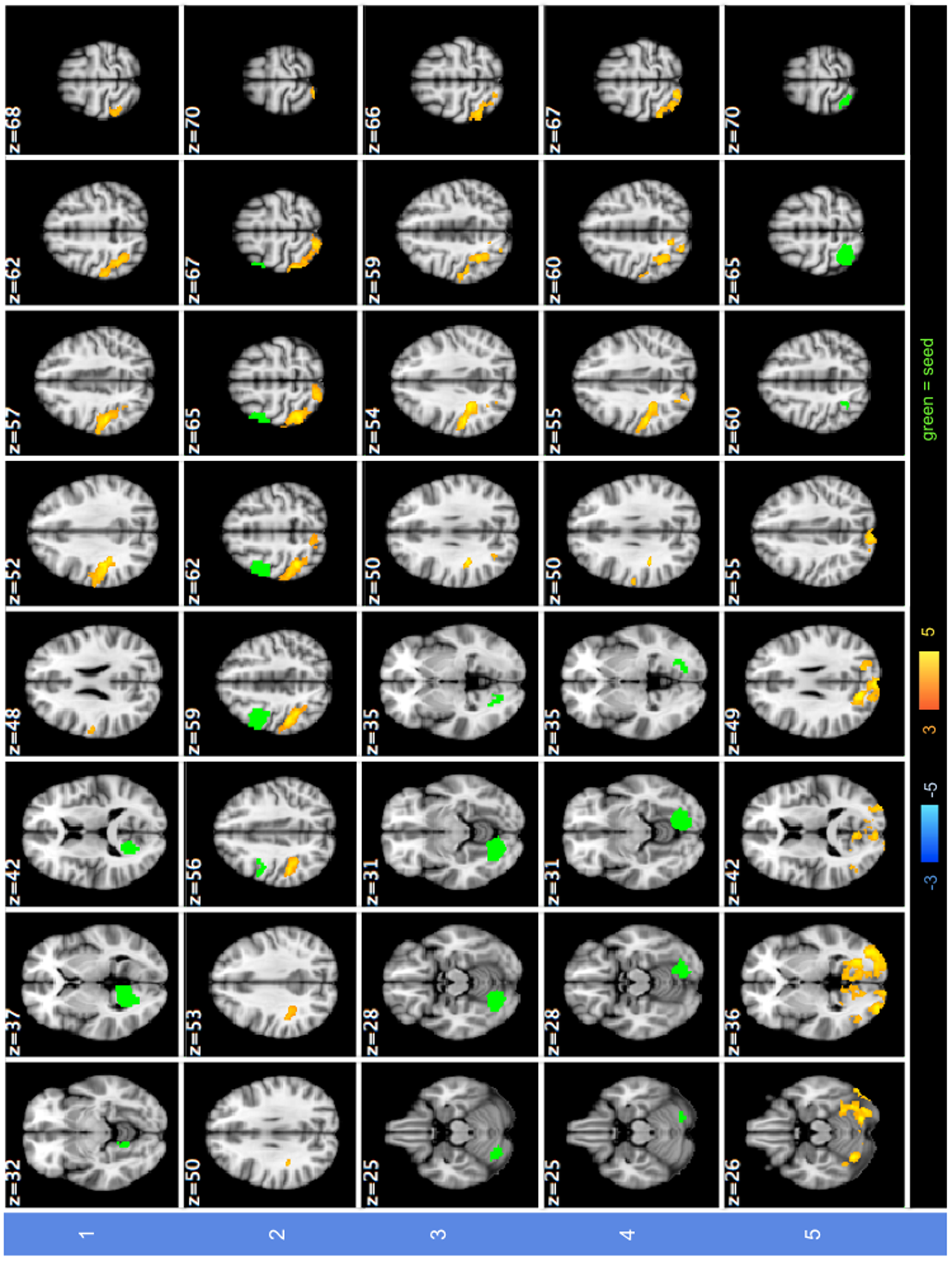

Supplement: Figure S4 — Axial slices of connections 1 to 5 correlated with NA. Details of the displayed connections can be found in Table 3. (TIF) [file pone.0068015.s004.tif]

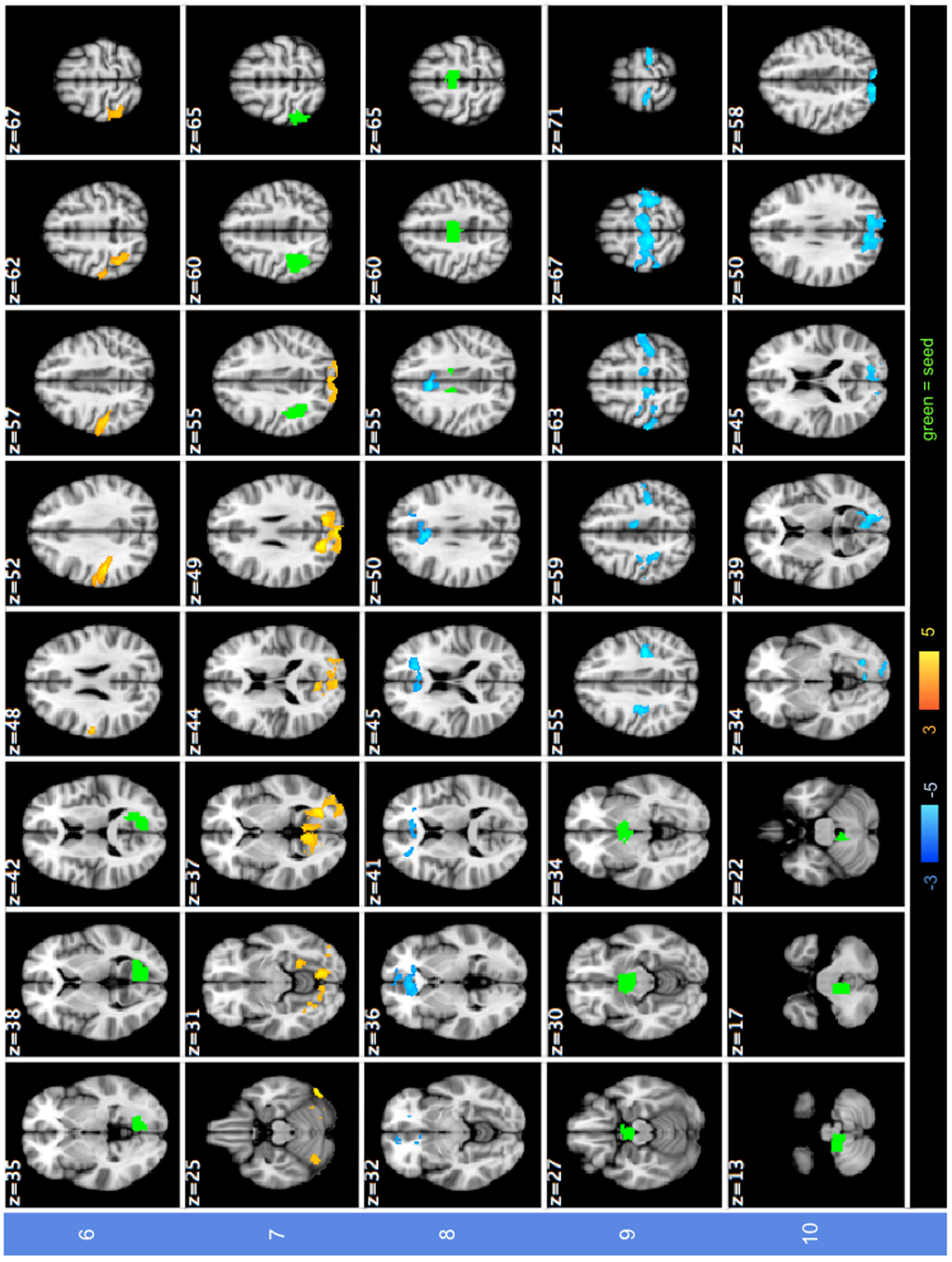

Supplement: Figure S5 — Axial slices of connections 6 to 10 correlated with NA. Details of the displayed connections can be found in Table 3. (TIF) [file pone.0068015.s005.tif]

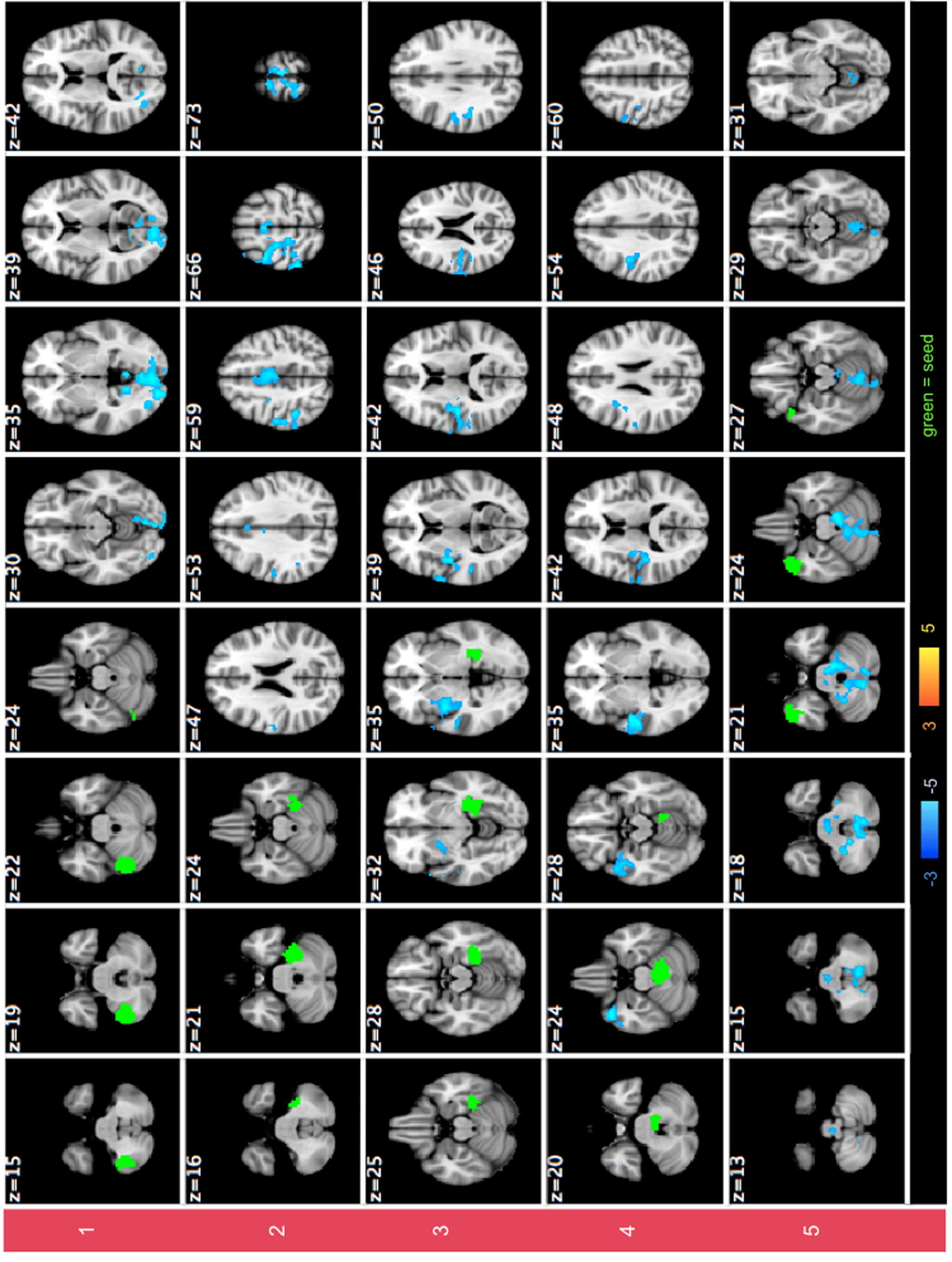

Supplement: Figure S6 — Axial slices of connections 1 to 5 correlated with PA. Details of the displayed connections can be found in Table 4. (TIF) [file pone.0068015.s006.tif]

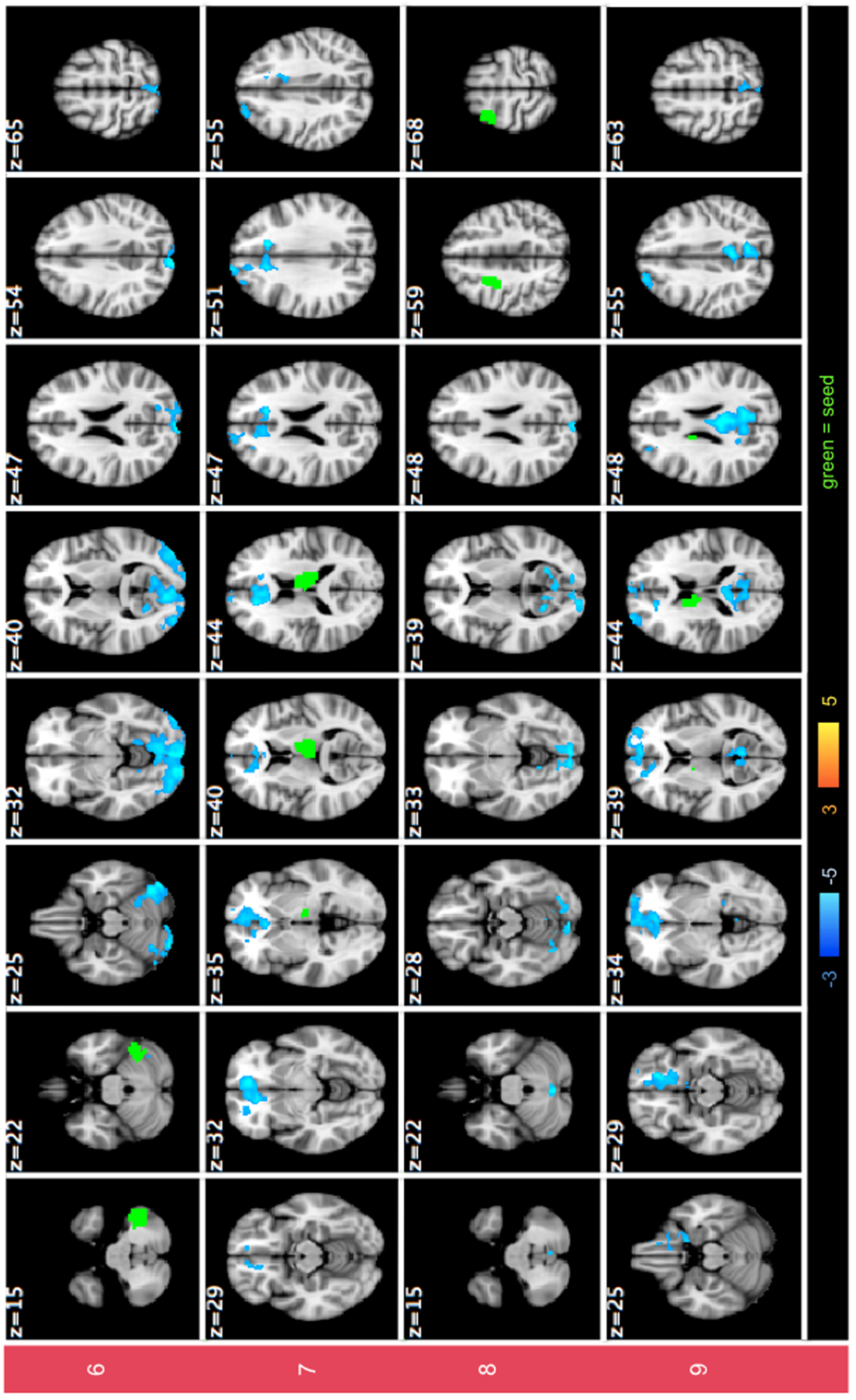

Supplement: Figure S7 — Axial slices of connections 6 to 9 correlated with PA. Details of the displayed connections can be found in Table 4. (TIF) [file pone.0068015.s007.tif]
