# Supplementary material for: Affect and the Brain's Functional Organization: A Resting-State Connectivity Approach
Source: PLoS One. 2013 Jul 23;8(7):e68015. doi: 10.1371/journal.pone.0068015 (PMC3720669; doi:10.1371/journal.pone.0068015)
Supplement: Table S1 — Details of additional connections at p<0.0005 for NA. (DOC) [file pone.0068015.s008.doc]

Supporting Table S1. Details of additional connections at p < 0.0005 for NA

|  | **Correlation with NA** | **Lat** | **Seed ROI** | **Lat** | **Connectivity Cluster** | **Cluster Size (mm^3^)** | **Cluster p-value** | **Peak Z value** | **x** | **y** | **z** | **Voxels LH in %** | **Voxels RH in %** | **LI** | **Domi-nance** |
| --- | --- | --- | --- | --- | --- | --- | --- | --- | --- | --- | --- | --- | --- | --- | --- |
| 1 | negative | R | OF, TF, ITG | BIL | pgACC, SFG, FP | 1796 | 0.000331 | 3.93 | 54 | 80 | 47 | 35.64 | 64.36 | -0.29 | R |
| 2 | positive | L | OL, PCN | BIL | LG, OF, OL, CRBL | 2028 | 0.000286 | 3.93 | 47 | 27 | 17 | 55.98 | 44.02 | 0.12 | BIL |
| 3 | positive | L | LG | R | SG, SPL | 1418 | 0.000252 | 3.81 | 25 | 50 | 52 | 41.43 | 58.57 | -0.17 | BIL |
| 4 | negative | R | MFG | L | adACC, dlPFC | 1253 | 0.000322 | 3.91 | 57 | 68 | 55 | 56.65 | 43.35 | 0.13 | BIL |
| 5 | negative | BIL | adACC | BIL | pdACC, M1 | 1595 | 0.000357 | 4.3 | 42 | 55 | 61 | 51.29 | 48.71 | 0.03 | BIL |

adACC=anterior dorsal anterior cingulate, CRBL=Cerebellum, dlPFC=dorsolateral prefrontal cortex, FP=frontal pole, Ins=Insula, LG=Lingual Gyrus, M1=Primary Motor Cortex, OF=Occipital Fusiform, OL=Lateral Occipital Complex, PCN=Precuneus, pdACC=posterior dorsal anterior cingulate, pgACC=perigenual anterior cingulate, SG=Supramarginal Gyrus, sgACC=subgenual anterior cingulate, TF=Temporal Fusiform, TP=temporal pole, MFG=middle frontal gyrus, SFG=superior frontal gyrus, SPL=superior parietal lobule

In order to assess the likelihood of false negatives, we lowered our threshold to p < 0.0005. Newly emerging connections typically replicated already observed patterns (for comparison, see Table 3 and Figure S1).
