# Supplementary material for: Affect and the Brain's Functional Organization: A Resting-State Connectivity Approach
Source: PLoS One. 2013 Jul 23;8(7):e68015. doi: 10.1371/journal.pone.0068015 (PMC3720669; doi:10.1371/journal.pone.0068015)
Supplement: Table S2 — Details of additional connections at p<0.0005 for PA. (DOC) [file pone.0068015.s009.doc]

***Supporting Table S2. Details of additional connections at p < 0.0005 for P***A

|  | **Correlation with PA** | **Lat** | **Seed ROI** | **Lat** | **Connectivity Cluster** | **Cluster Size (mm^3^)** | **Cluster p-value** | **Peak Z value** | **x** | **y** | **z** | **Voxels LH in %** | **Voxels RH in %** | **LI** | **Domi-nance** |
| --- | --- | --- | --- | --- | --- | --- | --- | --- | --- | --- | --- | --- | --- | --- | --- |
| 1 | negative | BIL | pgACC | BIL | Thalamus | 1180 | 0.000258 | 4.08 | 58 | 52 | 49 | 52.87 | 47.13 | 0.06 | BIL |
| 2 | negative | L | Caudate | BIL | PCC, PCN, Thalamus | 1667 | 0.000418 | 3.8 | 54 | 36 | 46 | 88.82 | 11.18 | 0.78 | L |

PCC=posterior cingulate, PCN=Precuneus, pgACC=perigenual anterior cingulate

In order to assess the likelihood of false negatives, we lowered our threshold to p < 0.0005. Newly emerging connections typically replicated already observed patterns (for comparison, see Table 4 and Figure S1).
