# Supplementary material for: Affect and the Brain's Functional Organization: A Resting-State Connectivity Approach
Source: PLoS One. 2013 Jul 23;8(7):e68015. doi: 10.1371/journal.pone.0068015 (PMC3720669; doi:10.1371/journal.pone.0068015)
Supplement: Table S3 — Details of additional connections at p<0.001 for NA. (DOC) [file pone.0068015.s010.doc]

***Supporting Table S3. Details of additional connections at p < 0.001 for N***A

|  | **Correlation with NA** | **Lat** | **Seed ROI** | **Lat** | **Connectivity Cluster** | **Cluster Size (mm^3^)** | **Cluster p-value** | **Peak Z value** | **x** | **y** | **z** | **Voxels LH in %** | **Voxels RH in %** | **LI** | **Domi-nance** |
| --- | --- | --- | --- | --- | --- | --- | --- | --- | --- | --- | --- | --- | --- | --- | --- |
| 1 | negative | R | OL, OP | BIL | pgACC, adACC, pdACC, FP | 1262 | 0.000992 | 3.83 | 41 | 76 | 44 | 30.11 | 69.89 | -0.4 | R |
| 2 | positive | BIL | OP, CN | R | SG, SPL | 1290 | 0.00076 | 3.79 | 26 | 47 | 51 | 30.76 | 69.24 | -0.38 | R |

adACC=anterior dorsal anterior cingulate, CN=Cuneal Cortex, FP=frontal pole, OL=Lateral Occipital Complex, OP= Occipital Pole, pdACC=posterior dorsal anterior cingulate, pgACC=perigenual anterior cingulate, SG=Supramarginal Gyrus, SPL=superior parietal lobule

In order to further assess the likelihood of false negatives, we lowered our threshold further to p < 0.001. Newly emerging connections typically replicated already observed patterns (for comparison, see Table 3 and Figure S2).
