# Supplementary material for: Affect and the Brain's Functional Organization: A Resting-State Connectivity Approach
Source: PLoS One. 2013 Jul 23;8(7):e68015. doi: 10.1371/journal.pone.0068015 (PMC3720669; doi:10.1371/journal.pone.0068015)
Supplement: Table S4 — Details of additional connections at p<0.001 for PA. (DOC) [file pone.0068015.s011.doc]

***Supporting Table S4. Details of additional connections at p < 0.001 for P***A

|  | **Correlation with PA** | **Lat** | **Seed ROI** | **Lat** | **Connectivity Cluster** | **Cluster Size (mm^3^)** | **Cluster p-value** | **Peak Z value** | **x** | **y** | **z** | **Voxels LH in %** | **Voxels RH in %** | **LI** | **Domi-nance** |
| --- | --- | --- | --- | --- | --- | --- | --- | --- | --- | --- | --- | --- | --- | --- | --- |
| 1 | negative | L | CRBL | BIL | SMA, M1, S1, SG, STS, STG, Ins, Put | 1962 | 0.000929 | 4.21 | 21 | 54 | 43 | 26.87 | 73.13 | -0.46 | R |
| 2 | negative | R | STS | BIL | CRBL | 1165 | 0.000815 | 4.08 | 51 | 45 | 23 | 26.04 | 73.96 | -0.48 | R |
| 3 | negative | L | PMC, SMA | BIL | CRBL | 1091 | 0.000791 | 4.09 | 47 | 28 | 20 | 89.15 | 10.85 | 0.78 | L |
| 4 | negative | L | Caudate, Put | BIL | PCC, PCN | 1343 | 0.000668 | 3.65 | 47 | 44 | 47 | 80.91 | 19.09 | 0.62 | L |

CRBL=Cerebellum, Ins=Insula, M1=Primary Motor Cortex, PCC=posterior cingulate, PCN=Precuneus, PMC=Premotor Cortex, Put=Putamen, S1=Primary Somatosensory Cortex, SMA=Supplementary Motor Area, STG=superior temporal gyrus, STS=superior temporal sulcus

In order to further assess the likelihood of false negatives, we lowered our threshold further to p < 0.001. Newly emerging connections typically replicated already observed patterns (for comparison, see Table 4 and Figure S2).
